# 11-[(*E*)-2-Fluoro­benzyl­idene]-8-(2-fluoro­phen­yl)-14-hy­droxy-6-thia-3,13-diaza­hepta­cyclo­[13.7.1.1^9,13^.0^2,9^.0^2,14^.0^3,7^.0^19,23^]tetra­cosa-1(22),15(23),16,18,20-pentaen-10-one

**DOI:** 10.1107/S1600536812025512

**Published:** 2012-06-13

**Authors:** Raju Suresh Kumar, Hasnah Osman, Abdulrahman I. Almansour, Suhana Arshad, Ibrahim Abdul Razak

**Affiliations:** a School of Chemical Sciences, Universiti Sains Malaysia, 11800 USM, Penang, Malaysia; b Department of Chemistry, College of Sciences, King Saud University, PO Box 2455, Riyadh 11451, Saudi Arabia; c School of Physics, Universiti Sains Malaysia, 11800 USM, Penang, Malaysia

## Abstract

In the title compound, C_34_H_26_F_2_N_2_O_2_S, an intra­molecular O—H⋯N hydrogen bond forms an *S*(5) ring motif. The piperidine ring adopts a chair conformation. The thia­zolidine ring and one of the pyrrolidine rings adopt envolope conformations with methyl­ene C atoms at the flap, whereas the other pyrrolidine ring adopts a half-chair conformation. The fluoro-substituted benzene rings form dihedral angles of 32.25 (10) and 38.27 (10)°, respectively, with the mean plane of the dihydro­acenaphthyl­ene ring system [maximum deviation = 0.043 (2) Å]. The dihedral angle between the fluoro-substituted benzene rings is 64.13 (14)°. In the crystal, mol­ecules are linked by weak C—H⋯O, C—H⋯F and C—H⋯S hydrogen bonds into a three-dimensional network.

## Related literature

For general background to the applications of nitro­gen heterocycles, see: Orru & de Greef (2003 ▶); Kirsch *et al.* (2004 ▶); Padwa (1984 ▶); For related structures, see: Kumar *et al.* (2010*a* ▶,*b* ▶, 2011*a* ▶,*b* ▶). For ring conformations, see: Cremer & Pople (1975 ▶). For hydrogen-bond motifs, see: Bernstein *et al.* (1995 ▶). For stability of the temperature controller used in the data collection, see: Cosier & Glazer (1986 ▶).
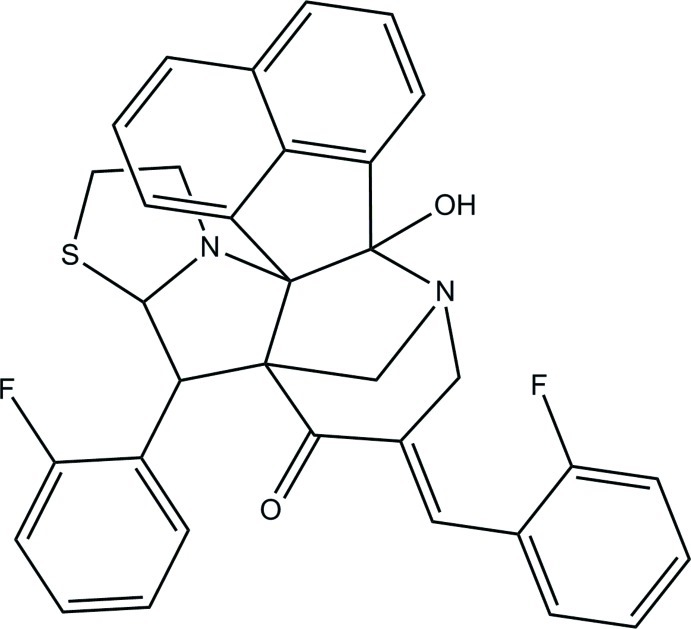


## Experimental

### 

#### Crystal data

C_34_H_26_F_2_N_2_O_2_S
*M* *_r_* = 564.63
Monoclinic, 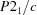

*a* = 11.1783 (10) Å
*b* = 16.1033 (14) Å
*c* = 15.2165 (13) Å
β = 92.838 (2)°
*V* = 2735.7 (4) Å^3^
*Z* = 4
Mo *K*α radiation
μ = 0.17 mm^−1^
*T* = 296 K
0.40 × 0.26 × 0.15 mm

#### Data collection

Bruker SMART APEXII DUO CCD area-detector diffractometer
Absorption correction: multi-scan (*SADABS*; Bruker, 2009 ▶) *T*_min_ = 0.936, *T*_max_ = 0.976
30643 measured reflections
7981 independent reflections
4714 reflections with *I* > 2σ(*I*)
*R*_int_ = 0.048

#### Refinement

*R*[*F* ^2^ > 2σ(*F* ^2^)] = 0.054
*wR*(*F* ^2^) = 0.173
*S* = 1.03
7981 reflections
374 parameters
H atoms treated by a mixture of independent and constrained refinement
Δρ_max_ = 0.44 e Å^−3^
Δρ_min_ = −0.37 e Å^−3^

### 

Data collection: *APEX2* (Bruker, 2009 ▶); cell refinement: *SAINT* (Bruker, 2009 ▶); data reduction: *SAINT*; program(s) used to solve structure: *SHELXTL* (Sheldrick, 2008 ▶); program(s) used to refine structure: *SHELXTL*; molecular graphics: *SHELXTL*; software used to prepare material for publication: *SHELXTL* and *PLATON* (Spek, 2009 ▶).

## Supplementary Material

Crystal structure: contains datablock(s) global, I. DOI: 10.1107/S1600536812025512/lh5479sup1.cif

Structure factors: contains datablock(s) I. DOI: 10.1107/S1600536812025512/lh5479Isup2.hkl

Supplementary material file. DOI: 10.1107/S1600536812025512/lh5479Isup3.cml

Additional supplementary materials:  crystallographic information; 3D view; checkCIF report

## Figures and Tables

**Table 1:** Hydrogen-bond geometry (Å, °)

*D*—H⋯*A*	*D*—H	H⋯*A*	*D*⋯*A*	*D*—H⋯*A*
O1—H1*O*1⋯N1	0.89 (3)	1.96 (3)	2.636 (2)	132 (3)
C14—H14*A*⋯O2^i^	0.97	2.54	3.156 (3)	121
C22—H22*A*⋯F1^ii^	0.93	2.44	3.351 (3)	166
C25—H25*A*⋯S1^iii^	0.93	2.78	3.545 (3)	140

Symmetry codes: (i) 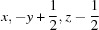; (ii) 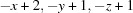; (iii) 
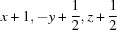
.

**Fig. 1.**
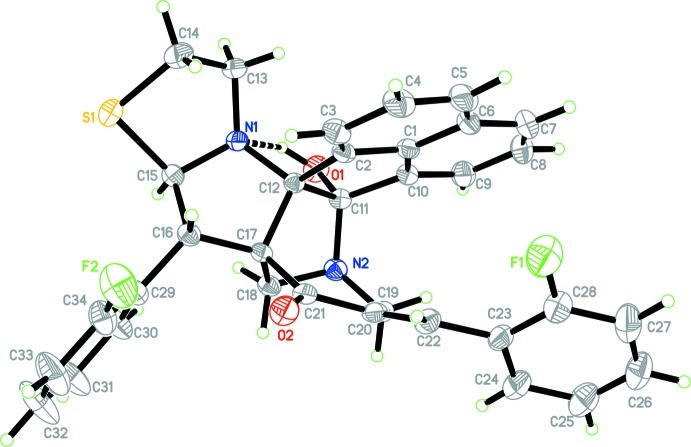
The molecular structure of the title compound, showing 30% probability displacement ellipsoids.

**Fig. 2.**
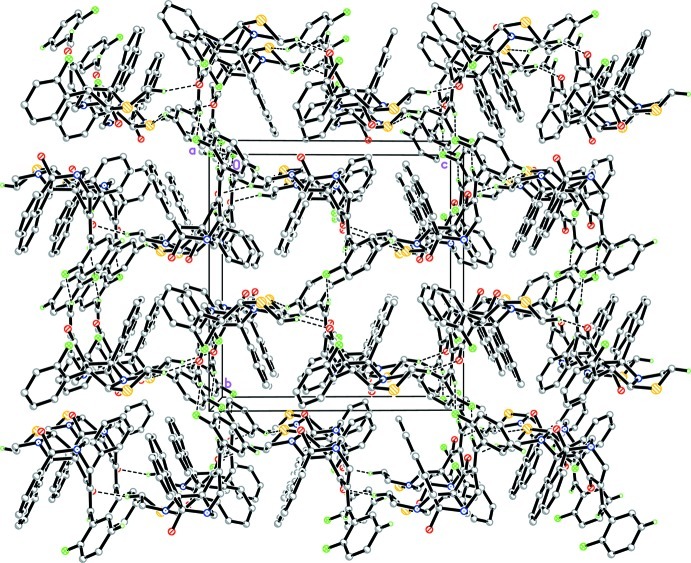
The crystal packing of the title compound. The H atoms not involved in the intermolecular interactions (dashed lines) have been omitted for clarity.

## supplementary crystallographic information

### Comment

The development of new efficient methods to synthesize nitrogen heterocycles with structural diversity is one of the major objectives of modern synthetic organic chemists as these heterocycles are widely prevalent in nature and play a pivotal role in the pharmaceutical and drug industry (Orru & de Greef, 2003; Kirsch *et al.*, 2004). 1,3-Dipolar cycloaddition of azomethine ylides is a versatile protocol for the construction of highly functionalized *N*-heterocycles (Padwa, 1984). As a continuation of our research program (Kumar *et al.*, 2010*a*,*b*; Kumar *et al.*, 2011*a*,*b*), we report the X-ray crystal structure determination of the title compound.

The molecular structure is shown in Fig. 1. The bond lengths and angles are within normal ranges and comparable to related structures (Kumar *et al.*, 2010*a*,*b*; Kumar *et al.*, 2011*a*,*b*). An intramolecular O1—H1O1···N1 hydrogen bond (Table 1) forms an *S*(5) ring motif (Bernstein *et al.*, 1995). The piperidine ring (N2/C17–C21) adopts a chair conformation with puckering parameters (Cremer & Pople, 1975), Q= 0.6115 (19) Å, Θ= 142.58 (18)° and Φ= 236.7 (3)°. For the thiazolidine ring, S1/N1/C13–C15 adopts an envelope conformation with atom C14 on the flap with puckering parameters Q= 0.4212 (19) Å and φ= 209.8 (3)°. The two pyrrolidine rings adopt different conformations, N1/C12/C15–C17 is twisted about C16–C17 bond [puckering parameters, Q= 0.3857 (19) Å and φ= 273.7 (3)°], hence adopting a half-chair conformation. Meanwhile, the N2/C11/C12/C17/C18 ring is in envelope conformation with atom C18 at the flap [puckering parameters Q= 0.4598 (19) Å and φ= 148.3 (2)°]. The fluoro-substituted benzene rings (C23–C28 & C29–C34) form dihedral angles of 32.25 (10) and 38.27 (10)°, respectively, with the mean plane of the dihydroacenaphthylene ring system [C2–C12, maximum deviation of 0.043 (2) Å at atom C11]. The dihedral angle between the fluoro substituted benzene rings is 64.13 (14)°.

In the crystal packing (Fig. 2), the molecules are linked into three dimensional network *via* intermolecular C14—H14A···O2^i^, C22—H22A···F1^ii^ and C25—H25A···S1^iii^ (Table 1) hydrogen bonds.

### Experimental

A mixture of 3,5-bis[(*E*)-(2-fluorophenyl) methylidene]-tetrahydro-4(1*H*)-pyridinone (1 mmol), acenaphthenequinone (1 mmol), and thiazolidine-2-carboxylic acid (1 mmol) were dissolved in methanol (5 ml) and refluxed for 1 h. After completion of the reaction as evident from TLC, the mixture was poured into water (50 ml). The precipitated solid was filtered and washed with water (200 mL) and recrystallized from ethyl acetate to give the title compound as colourless crystals.

### Refinement

The O-bound H atom was located from the difference map and refined freely, [O–H = 0.89 (4) Å]. The remaining H atoms were positioned geometrically [C–H = 0.93 and 0.98 Å] and refined using a riding model with *U*_iso_(H) = 1.2 *U*_eq_(C).

### Crystal data

**Table:**

C_34_H_26_F_2_N_2_O_2_S	*F*(000) = 1176
*M**_r_* = 564.63	*D*_x_ = 1.371 Mg m^−^^3^
Monoclinic, *P*2_1_/*c*	Mo *K*α radiation, λ = 0.71073 Å
Hall symbol: -P 2ybc	Cell parameters from 4640 reflections
*a* = 11.1783 (10) Å	θ = 2.5–22.5°
*b* = 16.1033 (14) Å	µ = 0.17 mm^−^^1^
*c* = 15.2165 (13) Å	*T* = 296 K
β = 92.838 (2)°	Block, colourless
*V* = 2735.7 (4) Å^3^	0.40 × 0.26 × 0.15 mm
*Z* = 4	

### Data collection

**Table:**

Bruker SMART APEXII DUO CCD area-detector diffractometer	7981 independent reflections
Radiation source: fine-focus sealed tube	4714 reflections with *I* > 2σ(*I*)
Graphite monochromator	*R*_int_ = 0.048
φ and ω scans	θ_max_ = 30.1°, θ_min_ = 1.8°
Absorption correction: multi-scan (*SADABS*; Bruker, 2009)	*h* = −15→15
*T*_min_ = 0.936, *T*_max_ = 0.976	*k* = −22→20
30643 measured reflections	*l* = −21→21

### Refinement

**Table:**

Refinement on *F*^2^	Primary atom site location: structure-invariant direct methods
Least-squares matrix: full	Secondary atom site location: difference Fourier map
*R*[*F*^2^ > 2σ(*F*^2^)] = 0.054	Hydrogen site location: inferred from neighbouring sites
*wR*(*F*^2^) = 0.173	H atoms treated by a mixture of independent and constrained refinement
*S* = 1.03	*w* = 1/[σ^2^(*F*_o_^2^) + (0.0842*P*)^2^ + 0.2935*P*] where *P* = (*F*_o_^2^ + 2*F*_c_^2^)/3
7981 reflections	(Δ/σ)_max_ < 0.001
374 parameters	Δρ_max_ = 0.44 e Å^−^^3^
0 restraints	Δρ_min_ = −0.37 e Å^−^^3^

### Special details

**Table:**

Geometry. All e.s.d.'s (except the e.s.d. in the dihedral angle between two l.s. planes) are estimated using the full covariance matrix. The cell e.s.d.'s are taken into account individually in the estimation of e.s.d.'s in distances, angles and torsion angles; correlations between e.s.d.'s in cell parameters are only used when they are defined by crystal symmetry. An approximate (isotropic) treatment of cell e.s.d.'s is used for estimating e.s.d.'s involving l.s. planes.
Refinement. Refinement of *F*^2^ against ALL reflections. The weighted *R*-factor *wR* and goodness of fit *S* are based on *F*^2^, conventional *R*-factors *R* are based on *F*, with *F* set to zero for negative *F*^2^. The threshold expression of *F*^2^ > σ(*F*^2^) is used only for calculating *R*-factors(gt) *etc*. and is not relevant to the choice of reflections for refinement. *R*-factors based on *F*^2^ are statistically about twice as large as those based on *F*, and *R*- factors based on ALL data will be even larger.

### Fractional atomic coordinates and isotropic or equivalent isotropic displacement parameters (Å^2^)

**Table:**

	*x*	*y*	*z*	*U*_iso_*/*U*_eq_	
S1	0.64031 (5)	0.09464 (4)	0.28495 (4)	0.05918 (19)	
F1	1.15043 (16)	0.49435 (9)	0.45961 (12)	0.0838 (5)	
F2	0.55699 (14)	0.25610 (10)	0.49142 (12)	0.0797 (5)	
O1	1.09128 (14)	0.06644 (8)	0.36084 (10)	0.0454 (3)	
O2	0.82349 (13)	0.30813 (9)	0.52734 (9)	0.0478 (4)	
N1	0.87293 (14)	0.12106 (9)	0.32117 (9)	0.0339 (3)	
N2	1.08274 (14)	0.13270 (10)	0.49723 (10)	0.0370 (4)	
C1	1.09271 (17)	0.27529 (11)	0.33216 (11)	0.0352 (4)	
C2	0.96866 (17)	0.26464 (11)	0.33643 (11)	0.0330 (4)	
C3	0.89385 (19)	0.32817 (12)	0.31060 (12)	0.0432 (5)	
H3A	0.8115	0.3233	0.3153	0.052*	
C4	0.9441 (2)	0.40161 (13)	0.27656 (14)	0.0527 (6)	
H4A	0.8935	0.4447	0.2581	0.063*	
C5	1.0638 (2)	0.41076 (13)	0.27015 (14)	0.0542 (6)	
H5A	1.0932	0.4593	0.2462	0.065*	
C6	1.1446 (2)	0.34828 (13)	0.29902 (13)	0.0454 (5)	
C7	1.2708 (2)	0.34971 (17)	0.30078 (16)	0.0612 (6)	
H7A	1.3095	0.3960	0.2792	0.073*	
C8	1.3370 (2)	0.28475 (18)	0.33346 (16)	0.0635 (7)	
H8A	1.4201	0.2877	0.3333	0.076*	
C9	1.28338 (19)	0.21281 (15)	0.36766 (14)	0.0513 (5)	
H9A	1.3302	0.1692	0.3901	0.062*	
C10	1.16086 (17)	0.20875 (12)	0.36704 (11)	0.0375 (4)	
C11	1.07658 (16)	0.14415 (11)	0.40053 (11)	0.0330 (4)	
C12	0.94494 (15)	0.18013 (10)	0.37635 (10)	0.0292 (3)	
C13	0.84768 (19)	0.13879 (14)	0.22702 (12)	0.0436 (5)	
H13A	0.8637	0.0899	0.1923	0.052*	
H13B	0.8988	0.1834	0.2083	0.052*	
C14	0.7179 (2)	0.16348 (16)	0.21350 (15)	0.0573 (6)	
H14A	0.6901	0.1559	0.1526	0.069*	
H14B	0.7062	0.2210	0.2299	0.069*	
C15	0.76423 (17)	0.09699 (12)	0.36640 (12)	0.0380 (4)	
H15A	0.7755	0.0416	0.3921	0.046*	
C16	0.75285 (16)	0.16069 (11)	0.44020 (11)	0.0346 (4)	
H16A	0.7184	0.2112	0.4135	0.042*	
C17	0.88387 (15)	0.17909 (11)	0.46585 (10)	0.0301 (4)	
C18	0.95895 (17)	0.11177 (12)	0.51626 (12)	0.0366 (4)	
H18A	0.9472	0.1144	0.5789	0.044*	
H18B	0.9380	0.0566	0.4950	0.044*	
C19	1.11878 (17)	0.20648 (12)	0.54978 (12)	0.0392 (4)	
H19A	1.1967	0.2248	0.5322	0.047*	
H19B	1.1270	0.1906	0.6113	0.047*	
C20	1.03218 (17)	0.27870 (12)	0.54077 (11)	0.0371 (4)	
C21	0.90487 (17)	0.26048 (12)	0.51384 (11)	0.0350 (4)	
C22	1.06286 (18)	0.35928 (13)	0.54605 (13)	0.0428 (5)	
H22A	1.0018	0.3974	0.5340	0.051*	
C23	1.18185 (19)	0.39387 (13)	0.56861 (13)	0.0456 (5)	
C24	1.2575 (2)	0.36255 (17)	0.63633 (16)	0.0631 (7)	
H24A	1.2343	0.3161	0.6676	0.076*	
C25	1.3663 (3)	0.3997 (2)	0.6574 (2)	0.0813 (9)	
H25A	1.4165	0.3774	0.7019	0.098*	
C26	1.4010 (3)	0.4689 (2)	0.6134 (2)	0.0853 (9)	
H26A	1.4743	0.4937	0.6285	0.102*	
C27	1.3281 (3)	0.50214 (18)	0.5467 (2)	0.0789 (8)	
H27A	1.3509	0.5494	0.5166	0.095*	
C28	1.2208 (2)	0.46361 (15)	0.52589 (16)	0.0564 (6)	
C29	0.67349 (17)	0.13516 (13)	0.51333 (13)	0.0415 (4)	
C30	0.6912 (2)	0.06281 (15)	0.56116 (15)	0.0575 (6)	
H30A	0.7529	0.0272	0.5471	0.069*	
C31	0.6191 (3)	0.0420 (2)	0.6296 (2)	0.0868 (10)	
H31A	0.6332	−0.0066	0.6615	0.104*	
C32	0.5273 (3)	0.0936 (2)	0.6497 (3)	0.1059 (13)	
H32A	0.4788	0.0797	0.6954	0.127*	
C33	0.5057 (3)	0.1652 (2)	0.6035 (2)	0.0877 (10)	
H33A	0.4429	0.2001	0.6169	0.105*	
C34	0.5792 (2)	0.18450 (16)	0.53672 (17)	0.0566 (6)	
H1O1	1.022 (3)	0.057 (2)	0.331 (2)	0.114 (12)*	

### Atomic displacement parameters (Å^2^)

**Table:**

	*U*^11^	*U*^22^	*U*^33^	*U*^12^	*U*^13^	*U*^23^
S1	0.0464 (3)	0.0822 (5)	0.0480 (3)	−0.0191 (3)	−0.0063 (2)	−0.0016 (3)
F1	0.0918 (12)	0.0607 (10)	0.0964 (12)	−0.0043 (8)	−0.0207 (10)	0.0173 (8)
F2	0.0590 (9)	0.0718 (10)	0.1096 (12)	0.0249 (8)	0.0175 (9)	0.0149 (9)
O1	0.0466 (8)	0.0346 (8)	0.0549 (8)	0.0092 (6)	0.0023 (7)	−0.0076 (6)
O2	0.0421 (8)	0.0472 (8)	0.0545 (8)	0.0072 (6)	0.0058 (6)	−0.0144 (7)
N1	0.0387 (8)	0.0350 (8)	0.0280 (7)	−0.0044 (6)	0.0009 (6)	0.0008 (6)
N2	0.0370 (8)	0.0394 (9)	0.0342 (7)	0.0033 (7)	−0.0008 (6)	0.0068 (6)
C1	0.0432 (10)	0.0354 (10)	0.0275 (8)	−0.0049 (8)	0.0057 (7)	−0.0020 (7)
C2	0.0434 (10)	0.0292 (9)	0.0266 (8)	−0.0018 (7)	0.0039 (7)	0.0020 (7)
C3	0.0514 (12)	0.0369 (11)	0.0414 (10)	0.0027 (9)	0.0038 (9)	0.0085 (8)
C4	0.0733 (16)	0.0354 (11)	0.0492 (12)	0.0010 (10)	0.0016 (11)	0.0115 (9)
C5	0.0793 (17)	0.0367 (11)	0.0467 (12)	−0.0131 (11)	0.0046 (11)	0.0098 (9)
C6	0.0571 (13)	0.0440 (12)	0.0357 (9)	−0.0151 (10)	0.0083 (9)	0.0009 (8)
C7	0.0607 (15)	0.0678 (16)	0.0559 (13)	−0.0264 (13)	0.0107 (11)	0.0060 (12)
C8	0.0410 (12)	0.0873 (19)	0.0629 (14)	−0.0206 (12)	0.0098 (11)	−0.0020 (13)
C9	0.0384 (11)	0.0634 (15)	0.0522 (12)	−0.0018 (10)	0.0043 (9)	0.0006 (11)
C10	0.0374 (10)	0.0438 (11)	0.0315 (9)	−0.0038 (8)	0.0050 (7)	−0.0025 (7)
C11	0.0355 (9)	0.0319 (9)	0.0316 (8)	0.0036 (7)	0.0017 (7)	0.0007 (7)
C12	0.0337 (8)	0.0270 (8)	0.0270 (7)	0.0004 (7)	0.0014 (6)	0.0022 (6)
C13	0.0502 (12)	0.0512 (12)	0.0292 (9)	−0.0080 (9)	−0.0011 (8)	0.0006 (8)
C14	0.0569 (14)	0.0693 (16)	0.0444 (11)	−0.0056 (11)	−0.0116 (10)	0.0096 (10)
C15	0.0405 (10)	0.0392 (11)	0.0344 (9)	−0.0067 (8)	0.0020 (7)	0.0036 (7)
C16	0.0358 (9)	0.0340 (10)	0.0341 (9)	0.0019 (7)	0.0031 (7)	0.0052 (7)
C17	0.0341 (9)	0.0301 (9)	0.0262 (7)	0.0000 (7)	0.0039 (6)	0.0039 (6)
C18	0.0403 (10)	0.0376 (10)	0.0319 (8)	0.0018 (8)	0.0028 (7)	0.0079 (7)
C19	0.0389 (10)	0.0474 (11)	0.0307 (8)	0.0025 (8)	−0.0041 (7)	0.0025 (8)
C20	0.0404 (10)	0.0449 (11)	0.0258 (8)	0.0005 (8)	0.0011 (7)	−0.0039 (7)
C21	0.0388 (10)	0.0394 (10)	0.0269 (8)	0.0014 (8)	0.0041 (7)	0.0011 (7)
C22	0.0426 (11)	0.0453 (12)	0.0404 (10)	0.0005 (9)	0.0003 (8)	−0.0070 (8)
C23	0.0452 (11)	0.0480 (12)	0.0434 (10)	−0.0060 (9)	0.0012 (9)	−0.0114 (9)
C24	0.0610 (15)	0.0730 (17)	0.0539 (13)	−0.0129 (12)	−0.0128 (11)	−0.0041 (12)
C25	0.0636 (17)	0.104 (2)	0.0739 (18)	−0.0186 (16)	−0.0236 (14)	−0.0036 (17)
C26	0.0626 (17)	0.100 (2)	0.091 (2)	−0.0331 (17)	−0.0146 (16)	−0.0061 (19)
C27	0.0731 (19)	0.0684 (19)	0.095 (2)	−0.0281 (15)	0.0043 (16)	0.0016 (16)
C28	0.0554 (14)	0.0524 (14)	0.0607 (14)	−0.0041 (11)	−0.0029 (11)	−0.0030 (11)
C29	0.0381 (10)	0.0470 (12)	0.0399 (10)	−0.0018 (8)	0.0071 (8)	0.0027 (8)
C30	0.0569 (14)	0.0564 (14)	0.0612 (14)	0.0016 (11)	0.0240 (11)	0.0149 (11)
C31	0.099 (2)	0.077 (2)	0.089 (2)	0.0018 (17)	0.0536 (18)	0.0274 (16)
C32	0.110 (3)	0.091 (2)	0.125 (3)	−0.001 (2)	0.085 (2)	0.017 (2)
C33	0.0672 (19)	0.082 (2)	0.119 (3)	0.0062 (15)	0.0536 (18)	−0.0048 (19)
C34	0.0436 (12)	0.0555 (14)	0.0718 (15)	0.0031 (10)	0.0148 (11)	0.0007 (12)

### Geometric parameters (Å, º)

**Table:**

S1—C14	1.804 (2)	C14—H14B	0.9700
S1—C15	1.8129 (19)	C15—C16	1.531 (3)
F1—C28	1.343 (3)	C15—H15A	0.9800
F2—C34	1.360 (3)	C16—C29	1.514 (3)
O1—C11	1.403 (2)	C16—C17	1.526 (2)
O1—H1O1	0.89 (4)	C16—H16A	0.9800
O2—C21	1.215 (2)	C17—C21	1.513 (2)
N1—C13	1.474 (2)	C17—C18	1.550 (2)
N1—C15	1.478 (2)	C18—H18A	0.9700
N1—C12	1.480 (2)	C18—H18B	0.9700
N2—C18	1.467 (2)	C19—C20	1.515 (3)
N2—C19	1.477 (2)	C19—H19A	0.9700
N2—C11	1.481 (2)	C19—H19B	0.9700
C1—C2	1.402 (3)	C20—C22	1.344 (3)
C1—C10	1.404 (3)	C20—C21	1.490 (3)
C1—C6	1.414 (3)	C22—C23	1.467 (3)
C2—C3	1.367 (3)	C22—H22A	0.9300
C2—C12	1.519 (2)	C23—C28	1.379 (3)
C3—C4	1.418 (3)	C23—C24	1.395 (3)
C3—H3A	0.9300	C24—C25	1.379 (4)
C4—C5	1.354 (4)	C24—H24A	0.9300
C4—H4A	0.9300	C25—C26	1.367 (4)
C5—C6	1.408 (3)	C25—H25A	0.9300
C5—H5A	0.9300	C26—C27	1.377 (4)
C6—C7	1.410 (3)	C26—H26A	0.9300
C7—C8	1.362 (4)	C27—C28	1.373 (4)
C7—H7A	0.9300	C27—H27A	0.9300
C8—C9	1.415 (3)	C29—C34	1.381 (3)
C8—H8A	0.9300	C29—C30	1.383 (3)
C9—C10	1.371 (3)	C30—C31	1.390 (3)
C9—H9A	0.9300	C30—H30A	0.9300
C10—C11	1.509 (3)	C31—C32	1.367 (4)
C11—C12	1.607 (2)	C31—H31A	0.9300
C12—C17	1.553 (2)	C32—C33	1.366 (5)
C13—C14	1.509 (3)	C32—H32A	0.9300
C13—H13A	0.9700	C33—C34	1.373 (4)
C13—H13B	0.9700	C33—H33A	0.9300
C14—H14A	0.9700		
			
C14—S1—C15	91.46 (9)	C29—C16—C15	115.48 (16)
C11—O1—H1O1	105 (2)	C17—C16—C15	101.78 (14)
C13—N1—C15	112.40 (15)	C29—C16—H16A	107.3
C13—N1—C12	119.96 (14)	C17—C16—H16A	107.3
C15—N1—C12	109.94 (14)	C15—C16—H16A	107.3
C18—N2—C19	108.21 (15)	C21—C17—C16	114.56 (15)
C18—N2—C11	103.11 (14)	C21—C17—C18	107.44 (14)
C19—N2—C11	115.84 (14)	C16—C17—C18	118.69 (15)
C2—C1—C10	114.11 (16)	C21—C17—C12	110.44 (13)
C2—C1—C6	122.82 (18)	C16—C17—C12	103.63 (13)
C10—C1—C6	123.02 (19)	C18—C17—C12	101.00 (13)
C3—C2—C1	119.18 (17)	N2—C18—C17	103.56 (14)
C3—C2—C12	132.01 (18)	N2—C18—H18A	111.0
C1—C2—C12	108.72 (15)	C17—C18—H18A	111.0
C2—C3—C4	118.8 (2)	N2—C18—H18B	111.0
C2—C3—H3A	120.6	C17—C18—H18B	111.0
C4—C3—H3A	120.6	H18A—C18—H18B	109.0
C5—C4—C3	121.9 (2)	N2—C19—C20	114.37 (15)
C5—C4—H4A	119.1	N2—C19—H19A	108.7
C3—C4—H4A	119.1	C20—C19—H19A	108.7
C4—C5—C6	121.4 (2)	N2—C19—H19B	108.7
C4—C5—H5A	119.3	C20—C19—H19B	108.7
C6—C5—H5A	119.3	H19A—C19—H19B	107.6
C5—C6—C7	128.3 (2)	C22—C20—C21	116.36 (17)
C5—C6—C1	115.9 (2)	C22—C20—C19	125.17 (18)
C7—C6—C1	115.7 (2)	C21—C20—C19	118.09 (17)
C8—C7—C6	121.3 (2)	O2—C21—C20	122.80 (17)
C8—C7—H7A	119.3	O2—C21—C17	121.94 (17)
C6—C7—H7A	119.3	C20—C21—C17	115.24 (15)
C7—C8—C9	122.0 (2)	C20—C22—C23	127.34 (19)
C7—C8—H8A	119.0	C20—C22—H22A	116.3
C9—C8—H8A	119.0	C23—C22—H22A	116.3
C10—C9—C8	118.5 (2)	C28—C23—C24	116.6 (2)
C10—C9—H9A	120.7	C28—C23—C22	120.2 (2)
C8—C9—H9A	120.7	C24—C23—C22	123.0 (2)
C9—C10—C1	119.31 (19)	C25—C24—C23	120.7 (3)
C9—C10—C11	132.13 (19)	C25—C24—H24A	119.7
C1—C10—C11	108.53 (16)	C23—C24—H24A	119.6
O1—C11—N2	108.43 (14)	C26—C25—C24	120.6 (3)
O1—C11—C10	112.41 (15)	C26—C25—H25A	119.7
N2—C11—C10	114.97 (15)	C24—C25—H25A	119.7
O1—C11—C12	110.40 (14)	C25—C26—C27	120.4 (3)
N2—C11—C12	105.62 (13)	C25—C26—H26A	119.8
C10—C11—C12	104.71 (14)	C27—C26—H26A	119.8
N1—C12—C2	116.72 (13)	C28—C27—C26	118.2 (3)
N1—C12—C17	103.90 (13)	C28—C27—H27A	120.9
C2—C12—C17	116.96 (14)	C26—C27—H27A	120.9
N1—C12—C11	111.35 (13)	F1—C28—C27	118.6 (2)
C2—C12—C11	103.80 (14)	F1—C28—C23	117.8 (2)
C17—C12—C11	103.44 (12)	C27—C28—C23	123.5 (2)
N1—C13—C14	108.68 (17)	C34—C29—C30	116.12 (19)
N1—C13—H13A	110.0	C34—C29—C16	121.05 (19)
C14—C13—H13A	110.0	C30—C29—C16	122.82 (18)
N1—C13—H13B	110.0	C29—C30—C31	121.7 (2)
C14—C13—H13B	110.0	C29—C30—H30A	119.2
H13A—C13—H13B	108.3	C31—C30—H30A	119.2
C13—C14—S1	104.09 (15)	C32—C31—C30	119.4 (3)
C13—C14—H14A	110.9	C32—C31—H31A	120.3
S1—C14—H14A	110.9	C30—C31—H31A	120.3
C13—C14—H14B	110.9	C33—C32—C31	120.9 (3)
S1—C14—H14B	110.9	C33—C32—H32A	119.6
H14A—C14—H14B	109.0	C31—C32—H32A	119.6
N1—C15—C16	105.43 (14)	C32—C33—C34	118.4 (3)
N1—C15—S1	107.77 (12)	C32—C33—H33A	120.8
C16—C15—S1	115.29 (14)	C34—C33—H33A	120.8
N1—C15—H15A	109.4	F2—C34—C33	117.8 (2)
C16—C15—H15A	109.4	F2—C34—C29	118.7 (2)
S1—C15—H15A	109.4	C33—C34—C29	123.5 (2)
C29—C16—C17	117.04 (15)		
			
C10—C1—C2—C3	175.02 (17)	S1—C15—C16—C29	79.94 (18)
C6—C1—C2—C3	−2.4 (3)	N1—C15—C16—C17	−33.48 (17)
C10—C1—C2—C12	−1.8 (2)	S1—C15—C16—C17	−152.20 (12)
C6—C1—C2—C12	−179.23 (16)	C29—C16—C17—C21	−73.8 (2)
C1—C2—C3—C4	2.8 (3)	C15—C16—C17—C21	159.33 (14)
C12—C2—C3—C4	178.72 (18)	C29—C16—C17—C18	54.9 (2)
C2—C3—C4—C5	−0.9 (3)	C15—C16—C17—C18	−71.94 (18)
C3—C4—C5—C6	−1.5 (4)	C29—C16—C17—C12	165.79 (15)
C4—C5—C6—C7	−176.7 (2)	C15—C16—C17—C12	38.94 (16)
C4—C5—C6—C1	1.9 (3)	N1—C12—C17—C21	−153.45 (14)
C2—C1—C6—C5	0.1 (3)	C2—C12—C17—C21	−23.2 (2)
C10—C1—C6—C5	−177.13 (18)	C11—C12—C17—C21	90.14 (15)
C2—C1—C6—C7	178.86 (18)	N1—C12—C17—C16	−30.30 (16)
C10—C1—C6—C7	1.7 (3)	C2—C12—C17—C16	99.91 (17)
C5—C6—C7—C8	177.9 (2)	C11—C12—C17—C16	−146.71 (14)
C1—C6—C7—C8	−0.7 (3)	N1—C12—C17—C18	93.09 (15)
C6—C7—C8—C9	−0.3 (4)	C2—C12—C17—C18	−136.70 (15)
C7—C8—C9—C10	0.4 (4)	C11—C12—C17—C18	−23.32 (16)
C8—C9—C10—C1	0.5 (3)	C19—N2—C18—C17	74.85 (16)
C8—C9—C10—C11	−177.5 (2)	C11—N2—C18—C17	−48.36 (17)
C2—C1—C10—C9	−179.01 (18)	C21—C17—C18—N2	−71.17 (17)
C6—C1—C10—C9	−1.6 (3)	C16—C17—C18—N2	156.89 (15)
C2—C1—C10—C11	−0.6 (2)	C12—C17—C18—N2	44.55 (16)
C6—C1—C10—C11	176.83 (16)	C18—N2—C19—C20	−51.43 (19)
C18—N2—C11—O1	−86.28 (16)	C11—N2—C19—C20	63.7 (2)
C19—N2—C11—O1	155.72 (15)	N2—C19—C20—C22	−148.31 (18)
C18—N2—C11—C10	146.96 (15)	N2—C19—C20—C21	24.3 (2)
C19—N2—C11—C10	29.0 (2)	C22—C20—C21—O2	−27.3 (3)
C18—N2—C11—C12	32.05 (17)	C19—C20—C21—O2	159.49 (17)
C19—N2—C11—C12	−85.94 (17)	C22—C20—C21—C17	151.05 (16)
C9—C10—C11—O1	−59.4 (3)	C19—C20—C21—C17	−22.2 (2)
C1—C10—C11—O1	122.42 (16)	C16—C17—C21—O2	−2.7 (2)
C9—C10—C11—N2	65.3 (3)	C18—C17—C21—O2	−136.84 (18)
C1—C10—C11—N2	−112.88 (17)	C12—C17—C21—O2	113.86 (19)
C9—C10—C11—C12	−179.3 (2)	C16—C17—C21—C20	178.97 (14)
C1—C10—C11—C12	2.55 (18)	C18—C17—C21—C20	44.81 (19)
C13—N1—C12—C2	11.7 (2)	C12—C17—C21—C20	−64.48 (18)
C15—N1—C12—C2	−120.95 (16)	C21—C20—C22—C23	−178.31 (18)
C13—N1—C12—C17	142.00 (16)	C19—C20—C22—C23	−5.6 (3)
C15—N1—C12—C17	9.40 (17)	C20—C22—C23—C28	141.7 (2)
C13—N1—C12—C11	−107.27 (18)	C20—C22—C23—C24	−42.4 (3)
C15—N1—C12—C11	120.13 (15)	C28—C23—C24—C25	−1.0 (4)
C3—C2—C12—N1	64.0 (3)	C22—C23—C24—C25	−177.1 (2)
C1—C2—C12—N1	−119.72 (16)	C23—C24—C25—C26	1.3 (5)
C3—C2—C12—C17	−59.9 (3)	C24—C25—C26—C27	−0.6 (5)
C1—C2—C12—C17	116.38 (16)	C25—C26—C27—C28	−0.3 (5)
C3—C2—C12—C11	−173.06 (19)	C26—C27—C28—F1	−178.4 (3)
C1—C2—C12—C11	3.20 (17)	C26—C27—C28—C23	0.6 (5)
O1—C11—C12—N1	1.75 (18)	C24—C23—C28—F1	179.1 (2)
N2—C11—C12—N1	−115.26 (15)	C22—C23—C28—F1	−4.7 (3)
C10—C11—C12—N1	122.96 (15)	C24—C23—C28—C27	0.1 (4)
O1—C11—C12—C2	−124.63 (15)	C22—C23—C28—C27	176.3 (2)
N2—C11—C12—C2	118.35 (14)	C17—C16—C29—C34	115.1 (2)
C10—C11—C12—C2	−3.43 (16)	C15—C16—C29—C34	−125.1 (2)
O1—C11—C12—C17	112.77 (15)	C17—C16—C29—C30	−64.0 (3)
N2—C11—C12—C17	−4.25 (17)	C15—C16—C29—C30	55.8 (3)
C10—C11—C12—C17	−126.03 (14)	C34—C29—C30—C31	−1.0 (4)
C15—N1—C13—C14	24.5 (2)	C16—C29—C30—C31	178.1 (3)
C12—N1—C13—C14	−107.0 (2)	C29—C30—C31—C32	0.9 (5)
N1—C13—C14—S1	−39.6 (2)	C30—C31—C32—C33	−0.1 (6)
C15—S1—C14—C13	35.33 (16)	C31—C32—C33—C34	−0.5 (6)
C13—N1—C15—C16	−121.28 (16)	C32—C33—C34—F2	−179.8 (3)
C12—N1—C15—C16	15.11 (18)	C32—C33—C34—C29	0.3 (5)
C13—N1—C15—S1	2.34 (19)	C30—C29—C34—F2	−179.5 (2)
C12—N1—C15—S1	138.73 (12)	C16—C29—C34—F2	1.4 (3)
C14—S1—C15—N1	−22.38 (15)	C30—C29—C34—C33	0.4 (4)
C14—S1—C15—C16	95.03 (15)	C16—C29—C34—C33	−178.8 (3)
N1—C15—C16—C29	−161.34 (15)		

### Hydrogen-bond geometry (Å, º)

**Table:**

*D*—H···*A*	*D*—H	H···*A*	*D*···*A*	*D*—H···*A*
O1—H1*O*1···N1	0.89 (3)	1.96 (3)	2.636 (2)	132 (3)
C14—H14*A*···O2^i^	0.97	2.54	3.156 (3)	121
C22—H22*A*···F1^ii^	0.93	2.44	3.351 (3)	166
C25—H25*A*···S1^iii^	0.93	2.78	3.545 (3)	140

Symmetry codes: (i) *x*, −*y*+1/2, *z*−1/2; (ii) −*x*+2, −*y*+1, −*z*+1; (iii) *x*+1, −*y*+1/2, *z*+1/2.
